# Comparison of visual and automated Deki Reader interpretation of malaria rapid diagnostic tests in rural Tanzanian military health facilities

**DOI:** 10.1186/s12936-018-2363-9

**Published:** 2018-05-29

**Authors:** Akili K. Kalinga, Charles Mwanziva, Sarah Chiduo, Christopher Mswanya, Deus I. Ishengoma, Filbert Francis, Lucky Temu, Lucas Mahikwano, Saidi Mgata, George Amoo, Lalaine Anova, Eyako Wurrapa, Nora Zwingerman, Santiago Ferro, Geeta Bhat, Ian Fine, Brian Vesely, Norman Waters, Mara Kreishman-Deitrick, Mark Hickman, Robert Paris, Edwin Kamau, Colin Ohrt, Reginald A. Kavishe

**Affiliations:** 1 National Institute for Medical Research, Tukuyu Centre, Tukuyu, Tanzania; 2 Henry Jackson Foundation Medical Research International, Dar es Salaam, Tanzania; 3 Tanzania Peoples Defense Forces, Dar es Salaam, Tanzania; 4 National Institute for Medical Research, Tanga Centre, Tanga, Tanzania; 5 Walter Reed Army Institute of Research, 503 Robert Grant Ave, Washington DC, USA; 6 Fio Corporation, Toronto, Canada; 7 FORGYN Health Systems Consultants LLc, Silver Spring, USA; 8 Kilimanjaro Christian Medical University College, Moshi, Tanzania; 9 Consortium for Health Action, Phnom Penh, Cambodia

**Keywords:** Malaria, Automated, Deki Reader, RDT, Interpretation

## Abstract

**Background:**

Although microscopy is a standard diagnostic tool for malaria and the gold standard, it is infrequently used because of unavailability of laboratory facilities and the absence of skilled readers in poor resource settings. Malaria rapid diagnostic tests (RDT) are currently used instead of or as an adjunct to microscopy. However, at very low parasitaemia (usually < 100 asexual parasites/µl), the test line on malaria rapid diagnostic tests can be faint and consequently hard to visualize and this may potentially affect the interpretation of the test results. Fio Corporation (Canada), developed an automated RDT reader named Deki Reader™ for automatic analysis and interpretation of rapid diagnostic tests. This study aimed to compare visual assessment and automated Deki Reader evaluations to interpret malaria rapid diagnostic tests against microscopy. Unlike in the previous studies where expert laboratory technicians interpreted the test results visually and operated the device, in this study low cadre health care workers who have not attended any formal professional training in laboratory sciences were employed.

**Methods:**

Finger prick blood from 1293 outpatients with fever was tested for malaria using RDT and Giemsa-stained microscopy for thick and thin blood smears. Blood samples for RDTs were processed according to manufacturers’ instructions automated in the Deki Reader. Results of malaria diagnoses were compared between visual and the automated devise reading of RDT and microscopy.

**Results:**

The sensitivity of malaria rapid diagnostic test results interpreted by the Deki Reader was 94.1% and that of visual interpretation was 93.9%. The specificity of malaria rapid diagnostic test results was 71.8% and that of human interpretation was 72.0%. The positive predictive value of malaria RDT results by the Deki Reader and visual interpretation was 75.8 and 75.4%, respectively, while the negative predictive values were 92.8 and 92.4%, respectively. The accuracy of RDT as interpreted by DR and visually was 82.6 and 82.1%, respectively.

**Conclusion:**

There was no significant difference in performance of RDTs interpreted by either automated DR or visually by unskilled health workers. However, despite the similarities in performance parameters, the device has proven useful because it provides stepwise guidance on processing RDT, data transfer and reporting.

## Background

Malaria case management, consisting of early diagnosis and prompt effective treatment, remains a vital component of malaria control and elimination strategies [1]. Since 2010, it has been recommended by the World Health Organization (WHO) that all cases of suspected malaria should have a parasitological test by either quality microscopy or a rapid diagnostic test (RDT) to confirm the diagnosis before treatment can be administered [1]. Failure to confirm malaria using recommended diagnostic techniques would lead to using anti-malarial drugs to treat patients who do not have malaria infection and ultimately contributing to the development of drug resistance [2]. Moreover, overdiagnosis is not cost-effective because the currently used artemisinin-based combination therapy (ACT) drugs are expensive. Ultimately, the correct diagnosis reduces the costs of case management in the health system and provides an opportunity to diagnose and properly manage other malaria-like febrile conditions.

For a long time, good quality microscopy has been regarded as a gold standard method for diagnosis and confirmation of malaria parasite infections [2, 3]. However, in many settings, especially low resourced environments, laboratory confirmation by microscopy for case management is impractical due to unavailability of laboratory facilities including skilled laboratory microscopists at the point-of-care [4–6]. Moreover, good performance of microscopy can be difficult to maintain because it is dependent on adequate training [7] and supervision of laboratory staff, electricity, good quality of reagents, staining processes and slides and of quality assurance [1, 8]. Additionally, whenever microscopy is used fully or minimally by improperly trained technicians, the data obtained has been shown to be dubious and unreliable [9]. Thus, RDTs are being advocated and used as alternative, or as an adjunct to microscopy at health facilities because they can be easily used by health workers with less training and equipment, and can be performed by non-specialists in remote settings [10, 11]. The RDTs provide fast test results for initiating or maintaining treatment of patients. However, for quality assurance, RDTs should be selected based on assessment results obtained using the WHO malaria RDT product testing programme [12, 13]. In many malaria endemic areas RDTs are being used with more frequently along with microscopy or in place of microscopy [3]. Microscopy detects or quantifies parasitaemia per volume of blood (asexual parasites/μl). The accuracy of microscopy relies mostly on expertise of microscopists in preparation, staining and reading the results of blood slides. On the other hand, RDTs qualitatively detect the presence of circulating antigens in blood. Most of the available RDTs can detect three different types of Plasmodium antigens; Plasmodium histidine rich protein 2 (PfHRP-2), Plasmodium lactate dehydrogenase (Pf-pLDH) and Plasmodium aldolase (Pf-pAldo). For the case of PfHRP2 based RDTs, false positives are not uncommon because of persisting HRP2 antigens in the blood after several weeks of successful treatment of infections, while in Pf-pLDH based RDT, pLDH is glycolytic enzymes which is quickly removed following successful treatment as previously reported [14, 15].

Although fast and simple in concept, RDT performance in practice requires health workers to be trained on aspects of RDT test performance so that they are able to correctly interpret results and properly record them [16]. Considering its cost effectiveness for correct malaria diagnosis in primary health facilities as compared to other methods, RDT have been recommended as a tool for malaria confirmation and consequently have been shown to improve management and care of patients [17, 18]. As such, there has been expansion of RDT usage worldwide which has aided management and care of malaria patients at health facilities of all levels [19]. For example, in 2013, approximately 319 million rapid diagnostic tests and 197 million slides were read and reported globally for the diagnosis and confirmation of malaria [20]. According to manufacturer sales data, RDTs sales increased from 46 million sold in 2008 to 314 million in 2014, and the number of diagnostic tests provided (RDTs and microscopy combined) exceeded the total number of courses of ACT administered in Africa [12].

In Tanzania, RDTs have become a solution for malaria diagnosis at rudimentary health care facilities such as dispensaries and health centres where microscopy is almost impossible [10]. Initially, RDTs were only used in research settings and this was followed by increased usage countrywide through a national roll-out campaign; from about two million in 2008 to 30 million RDTs in 2012 [21, 22]. Some common RDT brands that have been used in Tanzania include Paracheck^®^, ParaHIT, OptiMAL-IT^®^, SD Bioline and CareStart™. The SD Bioline Malaria Antigen Pf/pan (Standard Diagnostics, Hagal-Dong, Korea), that detects PfHRP2 (Pf) and pLDH (pan), is the RDT that has been recommended by the Tanzanian National Malaria Control Programme (NMCP) for malaria diagnosis countrywide since 2012 [23]. The introduction of RDTs in Tanzania has radically improved malaria diagnosis to the extent that both overuse of anti-malarials and proper management of patients have been improved [24, 25]. For example, the proportion of suspected malaria cases tested by RDT and treated in public health facilities increased from 58% in 2012 to 80% in 2016 [22].

Since RDTs are not able to detect parasitaemia infections at the lowest detection limit (usually < 100 asexual parasites/µl) which is the parasitaemia level commonly observed in low malaria transmission settings, more sensitive detection methods are required to accurately detect malaria. At very low parasitaemia, the RDT test line is usually very weak and can be falsely interpreted as a negative test as reported in various studies conducted in Tanzania [24, 26, 27]. Thus, despite their apparent simplicity, the preparation and interpretation of RDTs can be suboptimal and poor interpretation of test results can impact patient care. End-users of RDTs are often health workers located in remote areas, often volunteers with limited training hence they are likely to make mistakes in both RDT test preparation and interpretation of results. To help address this problem, Fio Corporation (Toronto, Canada) designed a Deki Reader™ (DR) to perform automated analysis and interpretation of RDTs. The DR is a ruggedized portable, universal reader of off-the-shelf lateral flow RDTs that provides a stable environment in which RDTs are imaged and results interpreted by on-board software. The device has been programmed to read most commonly used and commercially available histidine-rich-protein-2 (HRP2) based RDTs, including the SD Bioline malaria Ag Pf/pan (with both HRP2 and pLDH). Using a simple touch screen interface, an operator can enter patient identification number (ID), visually interpret RDT results, and input other patient information. This information is securely transmitted along with the RDT image and global positioning system (GPS) coordinates of the location to a centralized database over the local mobile phone network. All results from the field can be viewed from anywhere in the world in real-time by logging into a password protected portal using any web browser. Before validation of the DR for use in malaria detection, it was initially evaluated against traditional microscopy and polymerase chain reaction (PCR) in Colombia in South America and Tanzania in East Africa [28, 29]. The initial evaluation was done by research institutions that used skilled laboratory technologists to operate the device for interpreting RDTs. However, under field use where skilled laboratory technologists are not available, the device will not be operated by professional laboratory personnel but rather by unskilled health care workers. These are low cadre health care workers who have not attended any formal professional training in laboratory sciences; they are also known as laboratory attendants/auxiliary. Initially, they were recruited as laboratory cleaners and through on-the-job training they became laboratory attendants to carry out simple laboratory tests like RDT. Therefore, prior to scaling-up the use of the device for routine medical care in Tanzania that is facing shortages of skilled health care workers [30]—a comparison of performance of RDT was made between RDT analysis done by DR that is operated by less skilled health workers and human visual interpretation of RDT results against microscopy as a reference test. The sensitivity, specificity, positive predicative value (PPV), negative predicative value (NPV) and accuracy of the RDT was compared between automated analysis by DR and visual interpretation.

## Methods

### Study design and area

This cross-sectional study was conducted at Mgambo health facility in Tanga region. Tanga region located in north eastern Tanzania is a malaria endemic area with two main malaria transmission seasons every year, the high malaria transmission seasons (HMTS) and low malaria transmission seasons (LMTS). The HMTS starts from April and runs through August, which is during and immediately after long rain season (April to June), and the LMTS starts from October and runs through December, which is the short rain season of the year. The study was conducted during the end of the rainy season (from June to July, 2014) during the high malaria transmission period in Tanga region.

### Study populations

Study patients included military staff and civilians of all sexes and ages visiting the outpatient department at the military dispensary of Mgambo National Service Camp presenting with a fever (in the past 24 h) likely attributable to malaria. All outpatients attended the facility during HMTS in 2014 were eligible to participate to the study.

### Recruitment of study participants

Prior to recruitment, all outpatients or parents/guardian of sick children were informed about the study and requested to participate. They were asked for voluntary written informed consent prior to participation; two informed consent forms were filled by a participant or guardian and counter-signed by health personnel. One copy of the informed consent was kept within study file while the second copy was given to the participant. Thereafter, patients who gave informed consent were enrolled into the study.

### Training of data collectors

Three military laboratory attendants volunteered for and trained for 2 days in the preparation and interpretation of RDTs per the guidelines of the NMCP in place at the time when this study was conducted. These health care workers were further trained for 2 days on how to use the DR for malaria diagnosis. Additionally, two laboratory assistants (who had attended 1 year laboratory foundation course) who were employees of Tanzania Peoples defense forces at National Service camp, were trained for 1 week on preparation of thick and thin blood smears, fixing of thin smears and staining of the smears.

### Malaria diagnosis with Deki Reader of RDTs

In this study, 05fk60 SD Bioline Malaria Antigen Pf/pan (Standard Diagnostics Inc, Republic of Korea) RDT that detects PfHRP2 (*P. falciparum*) and pLDH (pan) was used for malaria diagnosis alongside microscopy. The DR provides automated sequential procedures to guide personnel on how to conduct the RDT test. The device prompts the user to insert the labeled RDT in a drawer for taking pictures of the RDT to recognize patient identification (ID) numbers before the test; thus, no patient names were entered in the device. After capturing an image and recognizing the brand of the RDT, the device prompts the user to remove the RDT from the device to proceed with taking blood samples from the patient. About 5 µl of blood sample was collected from a finger prick using a standard sampler (provided by the RDT manufacturer) and added to the blood well of the RDT cassette. Four drops of buffer were immediately added to the second well of the RDT cassette. After the incubation period (27 min for SD Bioline malaria Ag Pf/pan complying with manufacturer guidelines) as guided by the automated timer in the device, the user was prompted to insert the RDT in the device for the second time to capture an image of the RDT test which was then analysed and interpreted by the DR. The user was also prompted to input his/her own visual interpretation of the test results for both assays, i.e. *P. falciparum* and the pan-malaria line. The test results interpreted by the DR were not displayed on the device because the user was masked to the DR results. Both visual and device interpretation test results along with the image of the RDT and patient de-identified bio data were directly uploaded to the study web-portal.

### Malaria diagnosis using microscopy

From the same finger prick, thick and thin blood smears were prepared as guided by a standardized template; a slide with one frosted end was placed on top of the template and 6 μl of blood was immediately added to the slide on a large circle marked on the template which was then spread to cover the circle. Similarly, 2 μl blood was added at the edge of a small circle of the template for the thin film, which was then smeared as per standard operating procedures [31]. The prepared blood smears were air-dried, the thin smears were fixed by immersing in 100 ml beaker full of methanol Analar^®^, and both smears (thick and thin) were stained using 10% working solution of fresh Giemsa for 30 min. The stained smears were examined by experienced senior microscopists at Tanga Research Centre of the National Institute for Medical Research (NIMR) who was blinded of the results of the RDT interpreted visually and by the DR. To confirm positive films, asexual parasites were counted against 200 white blood cells (WBCs) while sexual parasites were counted against 500 WBCs. Assuming that each microlitre (µl) of blood contains 8000 WBCs parasites, densities were calculated by multiplying the parasite counts by 40 for asexual and 16 for sexual parasites. A smear was regarded to be negative after examining 200 high-power fields and not detecting parasites. Malaria species were detected and differentiated using thin blood smears depending on the appearance of the parasite, size and shape of the surrounding red cells, the presence of dots, stippling and pigment [31]. Other characteristics used for differentiation of the species included appearance of gametocytes and the developmental stages seen.

### Data management and analysis

Microscopy data were entered into Microsoft Access Data base. The data were double entered by two different data clerks, checked for consistency, validated and cleaned. Each patient’s record was identified by unique identification number which later was used to link microscopic data with patient’s de-identified demographic information and RDT results downloaded from secured web-portal. Data from RDT and the patients’ demographic information were entered by touching the screen interface in the DR and uploaded through mobile networks to a secured web-portal. The web-portal was accessed only by authorized project staff using password-protected. Data from RDT were downloaded to Microsoft Excel, checked, cleaned and then transferred to STATA software (Stata Corp, College Station, TX) for analysis. This data was merged with microscopy results, whereby all mismatched or uncorrected data were removed, including dropping of duplicate data to prepare the master file. Descriptive statistics with mean, standard deviations and proportions were used to summarize demographic information of the study participants Chi square was used to assess the associations between categorical variables. The sensitivity, specificity, positive predictive value (PPV), and negative predictive value (NPV) was used to compare automated RDT analysis and visual human interpretation against microscopy as reference test. A contingency table was used to calculate the indices where sensitivity as TP/TP+FN, specificity as TN/TN+FP, PPV as TP/TP+FP, NPV as TN/TN+FN and accuracy of the test as TP+TN/N. In these formulas N = number of individuals tested; TP = true positive (% of those who tested positive by both RDTs and microscopy; TN = true negative (% of those tested negative by RDTs and microscopy; FN = false negative (% of those who tested falsely as negative by RDTs while they actually were positive by microscopy; FP = false positive (% of those who tested falsely as positive by RDTs while they actually were negative by microscopy). The sensitivity was presented as proportion with 95% confidence intervals. P-value was considered significant at P < 0.05.

## Results

A total of 1293 among 1423 outpatients with axillary temperature ≥ 37.5 °C, reported fever in the past 24 h and/or other symptoms suggestive of malaria were recruited in the study. A total of 130 patients did not participate in this study of which 30 did not consent, 64 had no blood smears prepared together with RDTs and 36 had smears of low quality to carry out further analysis. The median age for study participants was 21 (Interquartile range 21–23) years (Table 1) and the majority of the participants (71.4%; 923/1293) were aged 18–24 years. More than two third (69.1%; 893/1293) of study participants were male.

**Table 1:** Demographics of participants and baseline diagnostic characteristics

Variable	
Number of participants, n	1293
Sex, n (%)	
Male	893 (69.1)
Female	400 (30.9)
Age (years)	
Median (IQR)	21 (20–23)
Age group (years), n (%)	
0–17	139 (10.8)
18–24	923 (71.4)
25+	231 (17.9)
RDT positivity rates n (%)	
By Deki Reader	777 (60.1)
Visual interpretation	769 (59.9)
Parasitaemia infection	
Microscopy positivity rate, n (%) [95% CI]	48.4 (45.7–51.2)
Parasite density range (asexual parasites/µl)	0–205, 120
GMPD [asexual parasites/µl (95% CI]	3399 (2872–4022)

CI, confidence interval at 95%; IQR, interquartile range; GMPD, geometric mean parasite density

Malaria RDT data were uploaded to portal by DR at speed of ≥ 76% of the records for a period of ≤ 24 h and only 26% of data were uploaded between 1 and 7 days. Malaria positivity rates by RDT were 60.1% (777/1293) and 59.9% (775/1293) as interpreted by DR and visually, respectively. Malaria positivity rate by microscopy as a reference test was 48.4% (626/1293) with a geometric mean parasite density of 3399 (95% CI = 2872–4022) asexual parasites/μl (Table 1).

*Plasmodium falciparum* was predominant species constituting 99.7% (624/626) of all malaria species found and two samples were mixed infections of *P. falciparum* and *Plasmodium malariae* (Table 2). Out of the 624 smear-positive *P. falciparum* infection samples, 5.8% (36/624) were falsely diagnosed as negative by automated DR and 6.4% (40/624) by visual interpretation (Table 3). Among the falsely diagnosed negative samples 5.0% (31/624) of all positives were the same samples diagnosed by both DR and visual interpretation. Out of two samples of mixed infections (*P. falciparum* and *P. malariae)*, in one smear positive sample *P. malariae* was falsely diagnosed as negative by both DR and visual interpretation. Out of the 667 smear negative samples, 28.2% (188/667) were falsely diagnosed as positive by DR and 27.6% (184/667) by visual interpretation (Table 3).

**Table 2:** Characterization of malaria plasmodium parasite species and antigens

Microscopy +ve		Deki Reader analysis					Visual interpretation				
		TP				FN	TP				FN
Species	No. (%)	Pan	Pf	Pf and pan	All		Pan	Pf	Pf and pan	All	
Pf	624 (48.3)	5	285	298	588 (93.9)	36 (5.8)	3	287	294	584 (93.6)	40 (6.4)
Po	0 (0)	0	0	0	0 (0)	0 (0)	0	0	0	0 (0)	0 (0)
Pm	0 (0)	0	0	0	0 (0)	0 (0)	0	0	0	0 (0)	0 (0)
Pf+Pm	2 (0.2)	0	1	0	1 (50.0)	1 (50.0)	0	1	0	1 (50.0)	1 (50.0)
Pf+Po	0 (0)	0	0	0	0 (0)	0 (0)	0	0	0	0 (0)	0 (0)
All	626 (48.4)	5	286	298	589 (94.1)	37 (5.9)	3	288	294	585 (93.5)	41 (6.5)

PT, true positive; FN, false negative; Pf, *P. falciparum*; Pm, *P. malariae*; Po, *P. ovale*

**Table 3:** Cross tabulation of RDT with microscopy test results, and performance of the RDT

RDT (any band)	Microscopy		Total	RDT performance
	Positive	Negative		
DR analysis				
Positive	589	188	777	Sensitivity = 589/626 * 100 = 94.1%
Negative	37	479	516	Specificity = 479/667 * 100 = 71.8%
Total	626	667	1293	PPV = 589/777 * 100 = 75.8%
				NPV = 479/516 * 100 = 92.8%
Visual interpretation				
Positive	585	184	769	Sensitivity = 585/626 * 100 = 93.5%
Negative	41	483	524	Specificity = 483/667 * 100 = 72.4%
Total	626	667	1293	PPV = 585/769 * 100 = 76.1%
				NPV = 483/524 * 100 = 92.2%

DR, Deki Reader; PPV, positive predictive value; NPV, negative predictive value

In reference to microscopy test as a gold standard, the sensitivity of RDTs in malaria diagnosis as interpreted by the device was 94.1% (95% CI 91.8–95.7) and that of human visual interpretation of RDTs was 93.5% (95% CI 91.6–95.5). The specificity of RDTs in malaria diagnosis as interpreted by the device was 71.8% (95% CI 67.3–74.3) and that of human visual interpretation of RDTs was 72.4% (95% CI 67.3–74.3). The PPV of RDT interpreted by the DR and human was 75.8% (95% CI 72.0–78.2) and 76.1% (95% CI 72.3–78.4), respectively. The NPV of RDT interpreted by the DR and interpreted visually were 92.8% (95% CI 89.9–94.7) and 92.2% (95% CI 89.8–94.6), respectively. The accuracy of RDT as interpreted by DR and visually was 82.6 and 82.1%, respectively. There was no significance difference between the PPV, NPV, the accuracy of RDT interpretation by automated DR and visual interpretation by a semi-skilled laboratory staff.

Table 4 shows that an increase in parasite density from < 200 parasites/μl to 4000 parasites/μl) was associated with increased sensitivity of RDT, from 69.2 to 100%, respectively; and the difference was statistically significant (P = 0.000). The trends were similar within and between RDT interpreted visually by human and by automated analysis.

**Table 4:** Comparison of sensitivity of RDT between automated analysis by Deki Reader and visual interpretation at different levels of parasite density

	Sensitivity of RDT; n/N (%)	
	Deki Reader interpretation	Visual interpretation
Parasite density (asexual parasites/µl)		
≥ 4001	325/327 (99.4)	325/327 (99.4)
2001–4000	67/67 (100)	67/67 (100)
801–2000	62/65 (95.4)	62/65 (95.4)
201–800	72/76 (94.7)	71/76 (93.4)
< 200	63/91 (69.2)	63/91 (69.2)
Statistical tests		
Chi square	122.1	119.1
P-value	0.000	0.000

## Discussion

In this study, automated analysis by DR and visual interpretation of RDTs by unskilled health care workers against microscopy was compared. This comparative study in interpreting RDTs between unskilled cadre of health workers and automated DR for routine outpatients is the first to be done in rural military health facilities. Whereas previous studies conducted in Tanzania, Colombia and Uganda [28, 29, 32] employed laboratory technicians from research institutions in performing RDT testing and operating the DR, in this study unskilled health care workers who had no formal training in laboratory sciences were employed to operate the automated device and also to visually interpret RDT test results. In the current trend of expanding the use of RDTs to the community level, community health workers are becoming solely responsible for malaria testing. Because of this, unskilled laboratory staff were chosen to operate the DR and visually interpret the RDT tests for comparative analysis.

In this study, the sensitivity, specificity, PPV, NPV, and accuracy RDTs as interpreted by the automated DR (94.1, 71.8, 75.8 and 82.6%) were similar to the interpretations which were visually done by unskilled health care workers (93.5, 72.4, 76.1 and 82.1%) with no significant differences. These findings are consistent with the previous studies conducted in Colombia, Tanzania and Uganda [28, 29, 32] where the interpretations of RDTs by automated device were comparable to expert visual interpretation (concordance rate > 95%) for *P. falciparum* which is the dominant malaria species in Tanzania.

Comparing this study with other recent studies that did not use DR for interpretation of test results in Tanzania, in this study the sensitivity of HRP2-based RDTs of about 94% which is lower than the > 95% recommended by the WHO [1]. Two studies conducted in Zanzibar, a constituent state of the United Republic of Tanzania observed a lower sensitivities of 79% [33] and 78.6% and higher specificity of 99.7% [33] compared to that reported in this study. Other studies done in Korogwe–Tanga of Tanzania [24, 34] also reported lower sensitivities of 88.6 and 88.9% and higher specificity of 88.2 and 97.4% respectively, compared to findings of this study. In 2008, in Rufiji and in Korogwe, investigators observed a sensitivity as low as 65% [35] and 88.9% [34], respectively. The sensitivity from this study (94.0%) is slightly lower than that reported in a study done in 2016 at multiple Tanzanian sites in Muheza, Ujiji, Muleba and Nachingwea where they observed sensitivity ranging from 97.3 to 99.3% at all sites [36] . A relative higher sensitivity (> 90%) for RDTs interpreted by both device and human coupled with high NPV in this study, is an indicative that the test is good for malaria diagnosis at point of care because very few patients will be missed by the test. Unfortunately in this study only one batch of RDT was used; it will be useful to test different batches in future studies to compare the performance of RDT for each batch.

The observed high sensitivity and low specificity of RDT should have been affected by high malaria transmission in the study area because high transmission is characterized by high false positive results and hence high sensitivity and low specificity as reported elsewhere [24]. In this regard and corresponding to late 2000s and early 2010s, when malaria transmission declined noticeably, the sensitivity of RDTs was low, but recently over the past 2 years, higher malaria transmission rates and parasite densities have been observed in Tanzania [36] and the sensitivity of RDTs has been observed to be a bit higher as reported in the recent studies [34, 36].

The reported specificity of about 72% found in this study is lower compared to what has been reported in other studies presumably due to the reported higher false positivity rate (28%), which could be due to persistence of PfHRP2 in the blood stream for several weeks after successful treatment leading clearance of parasites as reported elsewhere [15, 37]. Moreover, this study population constituted mostly (72%) by military recruits who were mostly coming from low malaria endemic areas and hence susceptible to infection in the study area which is highly endemic to malaria transmission. The high malaria prevalence in the area as demonstrated through high positivity rate by microscopy should have contributed to low specificity and high sensitivity of RDT as reported elsewhere [37, 38].

The sensitivity of RDT interpreted by automated DR and visually had a direct relationship with parasite density. The lower the parasite density (≤ 200 asexual parasites/µl) among blood samples, the lower the sensitivity of the RDT interpreted by the device (69.2%) and visually (69.2%), while the higher the parasite density (2001–4000 asexual parasites/µl) among blood samples, the higher the sensitivity (100%) of the RDT interpreted by the device and visually. At the highest parasite density level (≥ 4001 asexual parasites/µl), higher sensitivity (99.4%) was also registered although slightly lower by 0.6% compared to the maximum (100%) detection limit. This finding is in concordance with previous studies done in Tanzania [24, 39] that reported the direct proportionality of parasite density and prevalence with HRP2-based RDT. Other studies done in Nigeria [40] and Korea [41] also found that the sensitivity of RDT increased significantly (P < 0.0001) with an increase in *P. falciparum* parasitaemia. In contrary, in Central African Republic, although the tests performed less well in cases of low parasitaemia, they reported sensitivity of > 95% at > 500 parasites/μl [42] as opposed to sensitivity in this study (94.7%) at 201–800 parasites/μl. The sensitivity of RDT in relation to parasite densities as reported in this study samples are comparable to previous studies that reported in standardized categories of parasite density ranging from lower density (≤ 200 asexual parasites/μl) to higher density (≥ 2000 asexual parasites/μl) [43].

In this study, a false positivity rate of 28% was found by RDTs as interpreted by both the device and visually by unskilled health care workers. Such a high discordance rate could be attributed to the presence of persistent malaria antigens in the blood due to unreported recent self or hospital treatment with anti-malarials and hence leading to false positives which, has also been reported in other studies that used HRP2-based RDTs [44, 45]. The results of this study showed that ≤ 0.8% (5/626) of the samples were only detected by pan, while about 46% (286/626) were detected by Pf and ≥ 47% (298/626) by both Pf and Pan. Since pLDH detects both Pf and non-falciparum species, these results show that an RDT without pLDH would have missed less than 0.8% of the samples while pLDH missed over 45% of the samples, which were detected by the Pf band only. But since pLDH tests are usually combined with HRP2, these findings suggest that a combo test like SD Bioline is unlikely to miss significant number of parasites given the high sensitivity reported in this study.

In this regard, high false-positivity rate accompanied with low specificity can result into over diagnosis of malaria among non-malaria febrile patients. If a patient is found to be positive by HRP2 RDT simply due to history of recent malaria treatment, that result should be investigated more thoroughly and other possible causes of non-malaria fever should be investigated for proper treatment. However, the reported high false positive rate can be valid only if microscopy was performed very accurately (taking into account that PCR was not performed to correct microscopy results), otherwise, some reported false results in this study could be correct diagnosis as also reported in recent studies that RDTs are becoming more sensitive than microscopy performed inaccurately especially in low malaria transmission settings [46, 47]. Additionally, patients detected positive by RDT could have pure gametocytaemia that cannot be detected microscopically as studied elsewhere that presence of antigen secreting gametocytes is another possible cause of persistent HRP2 or pLDH antigenaemia [48]. Whilst, about 6% false negativity rate that was obtained could be due to deletion of *pfhrp*2 or/and *pfhrp*3 gene as also reported by other studies [49–51]. The reported false negative rate in this study could be also attributed by prozone effects as also reported by other studies [49]. In this study, it was observed that the weak test line on RDT sometimes could not be visualized by laboratory personnel with a decline in visual acuity and poor lighting systems in the testing room of these remote poor resource settings.

## Study limitations

Unlike other studies that employed polymerase chain reaction (PCR) for resolving discordant results of RDTs compared to that of microscopy, this study only used microscopy to confirm RDT results. Thus, dependence on microscopy to confirm RDT test results may have impacted these results because microscopy test results are likely to be affected by transmission intensity, prevalence of infections and parasite density as reported elsewhere [1].

## Conclusion

The performance agreement of the DR compared to visual interpretation conducted by unskilled health care workers in interpreting RDTs does suggest that the device can be used alone or concurrently with microscopy for malaria diagnosis. The use of this DR device at the point of care not only interprets RDT results, but has the added advantage to provide stepwise procedures for RDT testing, data transfer and timely reporting as also studied in Kenya and Uganda [32, 52]. Moreover, similar to skilled laboratory technologists, unskilled laboratory staff can be trained how to operate the DR for couple of days, assigned and use it for malaria diagnosis in remote areas, such as military camps; the DR is mostly automated in its operation and is, therefore, simple to use by any laboratory staff regardless level of education or professional training in laboratory technology. Therefore, it can be concluded that automated analysis of RDT by the DR operated by unskilled health care workers was similar to visual interpretation of RDT, but it has the added benefit by timely collecting surveillance data from rural settings and allowing managers to review RDT results from multiple rural sites simultaneously.
